# Supplementary material for: Associations between Coping Profile and Work Performance in a Cohort of Japanese Employees
Source: Int J Environ Res Public Health. 2022 Apr 15;19(8):4806. doi: 10.3390/ijerph19084806 (PMC9032047; doi:10.3390/ijerph19084806)
Supplement: Supplementary file 1 [file ijerph-19-04806-s001.zip › ijerph-1674418-supplementary.pdf]

Table S1. Association between coping profiles at baseline and work performance at follow-up using a regression model in complete case analysis (N = 1201).

	Bivariate model				Multivariate model			
	β	95% CI	t-value	p-value	β	95%CI	t-value	p-value
Problem-focused coping	2.16	(0.88, 3.45)	3.30	0.001	0.96	(-0.29, 2.22)	1.50	0.133
Emotion-focused coping	-0.43	(-1.87, 1.01)	-0.58	0.559	-0.61	(-1.98, 0.77)	-0.86	0.388
Dysfunctional coping	-2.44	(-3.56, -1.32)	-4.26	<0.001	-1.06	(-2.17, 0.05)	-1.87	0.062
PSS-10 score	-0.48	(-0.68, -0.28)	-4.65	<0.001	-0.33	(-0.53, -0.14)	-3.32	0.001

Adjusted for work performance, age, company, employment form, employment status, shift work, overworking hours/month, actual rest days /month, alcohol status, smoking status, exercise habits, and sleep duration at baseline. CI=confidence interval; PSS=Perceived Stress Scale

Table S2. Association between each coping profiles at baseline and work performance at follow-up using a regression model in complete case analysis (N = 1256).

	**β**	**95% CI**	**t-Value**	***p*-Value**
Brief COPE				
Problem-focused				
Active coping	-0.18	(-0.99, 0.62)	-0.44	0.658
Use of instrumental support	0.32	(-0.39, 1.03)	0.87	0.382
Planning	0.83	(0.02, 1.64)	2.01	0.044
Emotional-focused				
Acceptance	-0.10	(-0.81, 0.62)	-0.27	0.789
Use of emotional support	0.51	(-0.25, 1.27)	1.31	0.190
Humor	-0.16	(-0.82, 0.49)	-0.48	0.630
Positive reframing	-0.79	(-1.56, -0.01)	-1.98	0.048
Religion	0.49	(-0.35, 1.33)	1.14	0.254
Dysfunctional				
Behavioral disengagement	-0.07	(-0.82, 0.67)	-0.19	0.850
Denial	-0.62	(-1.49, 0.25)	-1.39	0.164
Self-blame	-1.24	(-1.86, -0.62)	-3.91	<0.001
Self-distraction	0.21	(-0.43, 0.86)	0.65	0.513
Substance use	0.38	(-0.21, 0.97)	1.27	0.206
Venting	-0.36	(-1.04, 0.31)	-1.07	0.286
PSS-10 score	-0.30	(-0.50, -0.09)	-2.85	0.004

Adjusted for work performance, age, company, employment form, employment status, shift work, overworking hours/month, actual rest days /month, alcohol status, smoking status, exercise habits, and sleep duration at baseline. CI=confidence interval; PSS=Perceived Stress Scale; Brief COPE = Brief Coping Orientation to Problems Experienced scale.
